# Analysis of the corrective contribution of strong halo-femoral traction in the treatment of severe rigid nonidiopathic scoliosis

**DOI:** 10.1186/s13018-020-02093-8

**Published:** 2020-11-30

**Authors:** Hongqi Zhang, Guanteng Yang, Chaofeng Guo, Jinyang Liu, Mingxing Tang

**Affiliations:** 1 Department of Spine Surgery and Orthopedics, Xiangya Hospital, Central South University, Changsha, China; 2 National Clinical Research Center for Geriatric Disorders, Xiangya Hospital, Central South University, No. 87, Xiangya Road, Changsha, 410008 Hunan China

**Keywords:** Severe rigid nonidiopathic scoliosis, Strong halo-femoral traction, Spinal fusion

## Abstract

**Introduction:**

Strong halo-femoral traction has been widely used in the field of severe rigid scoliosis correction. The objective of this study was to analyze the corrective contribution of strong halo-femoral traction in the treatment of severe rigid nonidiopathic scoliosis and discuss its meaning.

**Material and methods:**

A retrospective review was performed for patients with severe rigid nonidiopathic scoliosis who were treated with halo-femoral traction in our center from December 2008 to December 2015. All cases underwent halo-femoral traction for 2 to 4 weeks before a one-stage posterior operation, and the absolute and relative contribution rates of each orthopedic factor (bending, fulcrum, traction, surgery) were analyzed.

**Results:**

A total of 38 patients were included (15 males and 23 females), with a mean age of 16.4 ± 3.73 years (10–22 years) and follow-up of 55.05 ± 6.63 months (range 40–68 months). The etiology was congenital in 17 patients, neuromuscular in 14 patients, neurofibromatosis-1 in 3 patients, and Marfan syndrome in 2 patients. Congenital high scapular disease with scoliosis was found in 2 patients. The mean coronal Cobb angle of the major curve was 97.99° ± 11.47° (range 78°–124°), with a mean flexibility of 15.68% ± 6.65%. The absolute contribution rate (ACR) of bending was 27.26% ± 10.16%, the ACR of the fulcrum was 10.91% ± 2.50%, the ACR of traction was 32.32% ± 11.41%, and the ACR of surgery was 29.50% ± 9.70%. A significant difference in correction was noted between the ACRs of traction and the fulcrum (*P* < 0.05).

**Discussion:**

Strong halo-femoral traction plays a relatively significant role in the treatment of severe rigid nonidiopathic scoliosis while decreasing the risk of operation, and it is a safe and effective method for the treatment of severe rigid nonidiopathic scoliosis.

**Supplementary Information:**

The online version contains supplementary material available at 10.1186/s13018-020-02093-8.

## Introduction

Nonidiopathic scoliosis includes congenital scoliosis, neuromuscular scoliosis, neurofibromatosis-1, and Marfan syndrome, which usually presents as a known pathogeny with an early onset and rapid progression, leading to complex spinal deformities. Severe rigid nonidiopathic scoliosis is often associated with neural axis malformations, pulmonary dysfunction, and malnutrition, thus increasing the potential risk of a correction [1–4]. To decrease the risk of a correction, preoperational traction is applied in the treatment of nonidiopathic scoliosis.

Current methods of traction include halo-pelvic traction (HPT), halo-gravity traction (HGT), and halo-femoral traction (HFT). HPT provides a consistent force to the spine, but it causes severe trauma while placing the pins and is inconvenient for daily nursing and sleep. HGT is the most widely used traction method in clinical practice. During a long period of traction, the height of the thoracic spine and rib cage gradually lengthened, and the volume of the lungs also enlarged. Although patients have a good tolerance in HGT, since the procedure does not need patients to be absolutely lying in bed, the traction force of HGT is limited. In recent years, an increasing number of studies have focused on the treatment of complex spinal deformities via HFT preoperatively [5]. HFT can offer more powerful traction from both caudal and cephalic ends than the HGT. The simultaneous traction weight gradually enlarges the intervertebral space, resulting in alleviation of the angle of the main and secondary curve, thus improving the compliance of the spine and pulmonary function, which in return downsizes the deformity and helps avoid severe complications such as spinal cord injury. Additionally, preoperative improvement of the curve is proven to be beneficial for corrective surgery outcomes [4, 6–8].

However, to date, there are few reports on the treatment of severe rigid nonidiopathic scoliosis assisted by preoperative HFT. Hence, by reviewing a series of patients in our center and evaluating each parameter related to deformity correction, we aimed to assess the contribution of strong HFT to correction and the clinical value of HFT combined with one-stage posterior surgery in the treatment of severe rigid nonidiopathic scoliosis.

## Material and methods

### Clinical information

A retrospective review was performed for patients with severe rigid nonidiopathic scoliosis who were treated in our center between December 2008 and December 2015. All patients underwent physical and imaging examinations (X-ray, CT, MRI). All patients are reviewed in-person. The indications for preoperative HFT combined with one-stage posterior instrumentation and fusion were based on (1) a coronal Cobb angle of the major curve > 70° and bending flexibility < 30%, (2) no history of previous spinal surgery, (3) no preoperative neurologic symptoms, (4) a minimum of 2 years of follow-up, and (5) complete medical records and image data. The exclusion criteria were (1) had a history of spine surgery, (2) received anterior release, and (3) receiving three column osteotomies.

### Radiographic analysis

Standing long-cassette anteroposterior (AP) and lateral radiographs of the whole spine were taken at each point (before traction, after the operation and at the final follow-up). Supine long-cassette anteroposterior (AP) radiographs were taken each week after traction. Coronal and sagittal Cobb angles were measured on standing AP films, side bending films, and fulcrum-pushing films at each point (Additional file 1). The absolute contribution rate (ACR) of every corrective element was calculated by the following formula: MC: Major curve

$$ {\displaystyle \begin{array}{c}\mathrm{Bending}\ \mathrm{ACR}\left(\%\right)=\left(\mathrm{preoperative}\ \mathrm{MC}\ \mathrm{Cobb}\ \mathrm{angel}-\mathrm{Cobb}\ \mathrm{angle}\ \mathrm{on}\ \mathrm{bending}\ \mathrm{film}\right)/\left(\mathrm{preoperative}\ \mathrm{MC}\ \mathrm{Cobb}\ \mathrm{angel}-\mathrm{postoperative}\ \mathrm{MC}\ \mathrm{Cobb}\ \mathrm{angle}\right)\\ {}\mathrm{Fulcrum}\ \mathrm{ACR}\left(\%\right)=\left(\mathrm{Cobb}\ \mathrm{angle}\ \mathrm{on}\ \mathrm{bending}\ \mathrm{film}-\mathrm{Cobb}\ \mathrm{angle}\ \mathrm{on}\ \mathrm{fulcrum}\ \mathrm{film}\right)/\left(\mathrm{preoperative}\ \mathrm{MC}\ \mathrm{Cobb}\ \mathrm{angel}-\mathrm{postoperative}\ \mathrm{MC}\ \mathrm{Cobb}\ \mathrm{angle}\right)\\ {}\begin{array}{c}\mathrm{Traction}\ \mathrm{ACR}\left(\%\right)=\left(\mathrm{Cobb}\ \mathrm{angle}\ \mathrm{on}\ \mathrm{fulcrum}\ \mathrm{film}-\mathrm{Cobb}\ \mathrm{angle}\ \mathrm{after}\ \mathrm{traction}\right)/\left(\mathrm{preoperative}\ \mathrm{MC}\ \mathrm{Cobb}\ \mathrm{angel}-\mathrm{postoperative}\ \mathrm{MC}\ \mathrm{Cobb}\ \mathrm{angle}\right)\\ {}\mathrm{Surgery}\ \mathrm{ACR}\left(\%\right)=\left(\ \mathrm{cobb}\ \mathrm{angle}\ \mathrm{after}\ \mathrm{traction}-\mathrm{cobb}\ \mathrm{angle}\ \mathrm{after}\ \mathrm{surgery}\right)/\left(\mathrm{preoperative}\ \mathrm{MC}\ \mathrm{cobb}\ \mathrm{angel}-\mathrm{postoperative}\ \mathrm{MC}\ \mathrm{cobb}\ \mathrm{angle}\right)\end{array}\end{array}} $$

The relative contribution rate (RCR) of every corrective element was calculated by the following formula:

$$ {\displaystyle \begin{array}{c}\mathrm{Bending}\ \mathrm{RCR}\left(\%\right)=\left(\mathrm{preoperative}\ \mathrm{MC}\ \mathrm{Cobb}\ \mathrm{angel}-\mathrm{Cobb}\ \mathrm{angle}\ \mathrm{on}\ \mathrm{bending}\ \mathrm{film}\right)/\left(\mathrm{preoperative}\ \mathrm{MC}\ \mathrm{Cobb}\ \mathrm{angel}-\mathrm{postoperative}\ \mathrm{MC}\ \mathrm{Cobb}\ \mathrm{angle}\right)\\ {}\mathrm{Fulcrum}\ \mathrm{RCR}\left(\%\right)=\left(\mathrm{preoperative}\ \mathrm{MC}\ \mathrm{Cobb}\ \mathrm{angel}-\mathrm{Cobb}\ \mathrm{angle}\ \mathrm{on}\ \mathrm{fulcrum}\ \mathrm{film}\right)/\left(\mathrm{preoperative}\ \mathrm{MC}\ \mathrm{Cobb}\ \mathrm{angel}-\mathrm{postoperative}\ \mathrm{MC}\ \mathrm{Cobb}\ \mathrm{angle}\right)\\ {}\mathrm{Traction}\ \mathrm{RCR}\left(\%\right)=\left(\mathrm{preoperative}\ \mathrm{MC}\ \mathrm{Cobb}\ \mathrm{angel}-\mathrm{Cobb}\ \mathrm{angle}\ \mathrm{after}\ \mathrm{traction}\right)/\left(\mathrm{preoperative}\ \mathrm{MC}\ \mathrm{Cobb}\ \mathrm{angel}-\mathrm{postoperative}\ \mathrm{MC}\ \mathrm{Cobb}\ \mathrm{angle}\right)\end{array}} $$

### Traction protocol

Traction was started with a weight of 2 kg and gradually increased at a rate of 1 to 2 kg per day if the patients showed sufficient tolerance. According to the patients’ condition, the maximum traction weight was adjusted to 33–50% of the whole-body weight. Traction was maintained for a minimum of 18 h per day with the traction weight was unchanged at night if the patients did not complain of discomfort. During traction, the patient’s neurological status was frequently checked. If the Babinski sign, paresthesia, or any neurological compromise was noted, the weight was reduced immediately.

The active or passive joint exercise was performed to avoid rigid joints during the interval of traction, and pulmonary function training was also performed during traction. The length of the traction period was mainly determined by the radiographic evidence of curve improvement on weekly radiographs.

### Posterior instrumentation

Surgery was performed with halo-femoral traction intraoperatively. The patients were positioned prone maintaining halo-femoral traction with 1/3 of their body weight. All 38 patients were treated by hybrid constructs with screws and/or hooks. During the surgery, continuous neuro-monitorization was performed, and a wake-up test was performed after the final correction. Once the posterior surgery was performed, the halo-femoral apparatus was removed.

### Postoperative procedure

The drain was usually removed when the drainage flow was less than 30 ml/24 h. Patients were allowed to ambulate with a brace after remaining supine for 14 days postoperatively. The braces were continuously used for 6 to 8 months postoperatively. For better recovery, muscle strength training such as the leg raising straight sport and swimming is recommended.

### Evaluation of index and statistical analysis

The data were analyzed using SPSS (version 25.0, SPSS Inc.). Paired *t* tests were used to compare parameters at the preoperative, postoperative, and final follow-up stages. A *p* value < 0.05 indicated a statistically significant difference.

## Results

### Patient characteristics

A total of 38 patients were included (15 males and 23 females, mean age 16.4 ± 3.73 years, range 10–22 years) with a mean follow-up period of 55.05 ± 6.63 months (range 40–68 months). 27 patients were living in rural area while the other 11 patients were living in city. The etiology was congenital in 17 patients, neuromuscular in 14 patients, neurofibromatosis-1 in 3 patients, and Marfan syndrome in 2 patients. Congenital high scapular disease with scoliosis was found in 2 patients (Table 1). The mean operation time was 4.83 ± 0.79 h, and the average days with maximum weight traction were 18.26 ± 2.43 days (14–23 days). The average maximum traction weight was 18.23 kg ± 1.99 kg, which was equal to 46.46.2% ± 1.99% (range 36–54.9%) of the patients’ total body weight.

**Table 1:** Demographic of study populations

General information	cases *n* = 38
Gender	
Male	15 (60.52%)
Female	23 (69.56%)
Diagnosis	
Congenital scoliosis	17 (44.73%)
Neuromuscular scoliosis	14 (36.84%)
Neurofibromatosis-1 scoliosis	3 (7.89%)
Marfan syndrome with scoliosis	2 (5.26%)
Congenital high Scapular disease with scoliosis	2 (5.26%)
Traction related complication	
Brachial plexus palsy	1 (2.63%)
Pin tract infection	1 (2.63%)
Deep vein thrombosis	1 (2.63%)
Femoral nerve palsy	1 (2.63%)
Rigid knee/hip	2 (5.26%)
Patient resides	
Rural	27 (71.05%)
City	11 (28.94%)

### Radiographic analysis

**Fig. 1:**
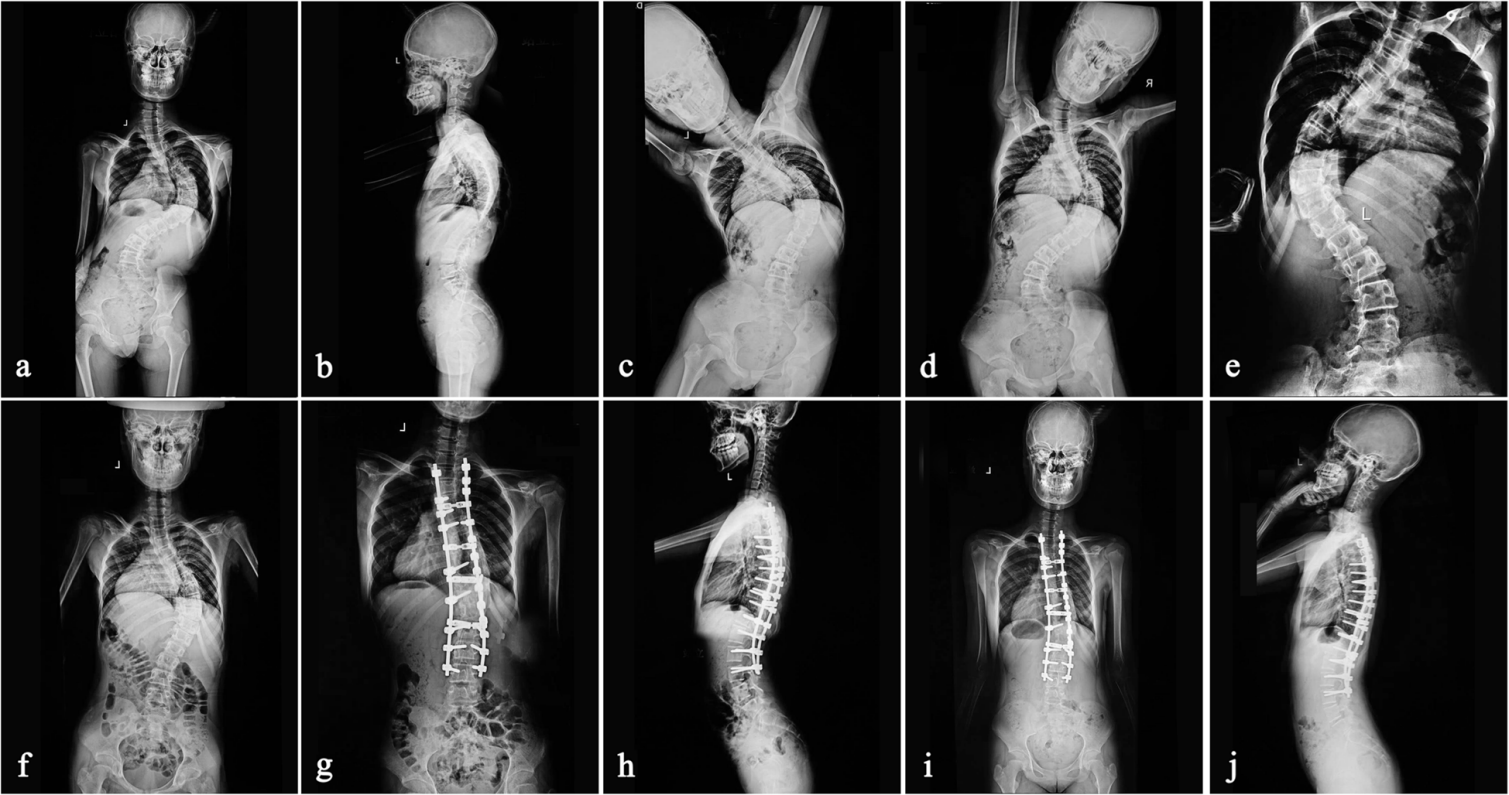
A 15-year-old female patient with Marfan syndrome had a scoliosis of 92° (**a**) and a kyphosis of 76° (**b**). Major coronal curve decreased to 80° (**d**) at side bending radiograph and then decreased to 72° (**e**) at fulcrum radiograph. After a 3-week traction, scoliosis was corrected to 58° (**f**). Postoperative standing radiograph demonstrated scoliosis was corrected to 24° (**g**), and kyphosis to 18° (**h**), and the 2-year follow-up shows the scoliosis was 26° (**i**), and kyphosis to 20° (**j**)

The average preoperative major curve magnitude was 97.99° ± 11.47° (range 78–124°), which decreased to 83.00° ± 14.0° (range 58–106°) on side bending and then reduced to 76.97° ± 14.31° (range 50–100°) on the fulcrum film. The major curve averaged 59.53° ± 12.02° (range 39–82°) at the end of the halo-femoral traction treatment and then improved to 44.42° ± 12.09° (range 26–72°) after posterior corrective surgery. The mean Cobb angle at final follow-up was 46.73° ± 12.46° (range 28–79°). The average preoperative kyphosis angle was 66.29° ± 13.51° (range 51–107°), which decreased to 31.71° ± 7.52° (range 21–50°) postoperatively and 33.76° ± 7.68° (range 22–52°) at the final follow-up (Fig. 1). The average preoperative C7 plumb line to center sacral vertical line (C7-CSVL) was 12.5 ± 3.8 mm, which decreased to 6.94 ± 4. 6 mm postoperatively and 7.26 ± 5.8 mm at the final follow-up. The average preoperative sagittal vertebrae axis (SVA) was 28.4 ± 15.8 mm, which decreased to 21.2 ± 9.6 mm postoperatively and 23.4 ± 10.5 mm at the final follow-up (Table 2).

**Table 2:** Radiographic data of study populations

General information	
The cobb angel of the major curve	
AP film	97.99° ± 11.47°
Bending film	83.00° ± 14.0°
Fulcrum film	76.97° ± 14.31°
After traction film	59.53° ± 12.02°
Postoperation film	44.42° ± 12.09°
Final follow-up film	46.73° ± 12.46°
The kyphosis angle of the major curve	
Preoperation film	66.29° ± 13.51°
Postoperation film	31.71° ± 7.52°
Final follow-up film	33.76° ± 7.68°
C7-CSVL	
Preoperation	12.5 ± 3.8 mm
Postoperation	6.94 ± 4.6 mm
Final follow-up	7.26 ± 5.8 mm
SVA	
Preoperation	28.4 ± 15.8 mm
Postoperation	21.2 ± 9.6 mm
Final follow-up	23.4 ± 10.5 mm

### Correction rate-related factors

The absolute contribution of bending to correction was 27.26% ± 10.16%, the absolute contribution of the fulcrum to correction was 10.91% ± 2.50%, the absolute contribution of traction to correction was 32.32% ± 11.41%, and the absolute contribution of surgery to correction was 29.50% ± 9.70%. A significant difference in correction was noted between the absolute contribution rate of traction and the absolute contribution rate of the fulcrum (*p* < 0.05), and there was no significant difference when comparing the absolute contribution rate of traction to that of bending or surgery (*p* > 0.05) (Table 3).

**Table 3:** Absolute and relative contribution rate of each factor

	Bending contribution rate	Fulcrum contribution rate	Traction contribution rate	Surgery contribution rate
Absolute	27.26% ± 10.16%	10.91% ± 2.50%	32.32% ± 11.41%^*^	29.50% ± 9.70%
Relative	27.26% ± 10.16%	38.17% ± 11.57%	74.05% ± 14.77%^#*^	–

^#^Compared with bending contribution rate, *P* < 0.05

^*^Compared with fulcrum contribution rate, *P* < 0.05

The relative contribution of bending to correction was 27.26% ± 10.16%, the relative contribution of the fulcrum to correction was 38.17% ± 11.57%, and the relative contribution of traction to correction was 74.05% ± 14.77%. There was a significant difference when comparing the relative contribution rate of traction to the bending or fulcrum relative contribution rates (*p* < 0.05) (Table 3).

### Complications

During traction, 1 patient experienced brachial plexus palsy, and 1 patient experienced femoral nerve palsy. The symptoms disappeared after the removal of the increased weight. One patient suffered from deep vein thrombosis (DVT), and the patient then underwent inferior vena cava filter placement. A pin infection occurred in 1 patient and was controlled by debridement. Two patients developed a rigid knee/hip due to fear of moving the knee/hip. The total incidence of traction complications was 15.8% (Table 1).

## Discussion

HGT has been reported to successfully assist in the management of spinal deformities [9–11]. However, due to the limited traction weight, the efficiency of treating severe rigid scoliosis is debated. Yang et al. [12] systematically reviewed a total of 351 severe spinal deformity patients treated with HGT preoperatively, and the patients did not have better correction postoperatively. Koller et al. [13] reported that HGT did not significantly improve severe curves without prior anterior or posterior surgical release. Sponseller et al. [14] found that HGT did not increase the main coronal curve or sagittal plane correction in a multicenter, retrospective, nonrandomized comparison study.

Compared to HGT, which has a limited traction weight, HFT can offer stronger and simultaneous traction forces [15, 16]. In our study, the mean preoperative major curve was 97.99° ± 11.47°, with a mean flexibility of 15.68% ± 6.65%, and it had only a 6.23% ± 1.79% improvement on the fulcrum film; however, after a mean 18.15 ± 2.01 kg (46.46% ± 5.36% of body weight) maximum traction weight for 18.26 ± 2.43 days, the average correction rate added 17.83% ± 5.41%, reaching a total of 39.74% ± 6.22% at the end of HFT. Similarly, Wang et al. [7] reported 21 cases with extremely severe rigid scoliosis treated by HFT before posterior vertebral column resection. The mean preoperative major curve was 153°, and after 4 weeks of traction, the mean decrease in Cobb angle achieved a 33.7% correction of scoliosis. The advantages of strong HFT could be three-fold: first, strong HFT can offer patients more effective traction time, and traction effects tend to be better, especially when applied during sleep at night to weak muscles. Thus, through continuous heavy traction, paravertebral soft tissue and intervertebral space not only in the area of the major curve but also in the second curve can be released. Second, severe rigid nonidiopathic scoliosis is often associated with neural axis malformations. The gradually increased corrective force contribution helps the surgeon assess the tolerance of the spinal cord, which helps to achieve adequate reduction and optimum balance. Moreover, during the traction period, patients can improve their pulmonary function and malnutrition states, thus increasing their tolerance of the operation and reducing hospital stays and costs.

Another advantage of HFT is that the patients will maintain HFT during surgery, and intraoperative halo-femoral traction leads to apical vertebral de-rotation. This de-rotation of the spine facilitates surgical exposure and screw rod insertion and limits the force on implants [17, 18]. In the present study, we also performed intraoperative HFT during the posterior surgery, and the Cobb angle improved to 42.56° ± 11.63° after posterior corrective surgery. The average correction rate obtained was 59.5% ± 8.5% without any postoperative neurological complications.

In our study, the absolute contribution rate of bending was 27.26% ± 10.16%, the absolute contribution rate of the fulcrum was 10.91% ± 2.50%, the absolute contribution rate of traction was 32.32% ± 11.41%, and the absolute contribution rate of surgery was 29.50% ± 9.70%. In terms of the first-level corrective force, the Cobb angle improvement of bending is relatively easy to obtain, while for the second-level corrective force, i.e., that of the fulcrum, the Cobb angle improvement is relatively demanding to obtain because only with a vertical push force at the coronal parietal region can this measurement be effective. Traction, as the third-level corrective force, is difficult to obtain. Our results show that both the second level and the third level of corrective force were obtained after an average of 18.26 ± 2.43 days of HFT. The average correction after traction was an average of 39.74% ± 6.22%, which was a significant improvement compared with the correction obtained from the side fulcrum film in our study (*p* < 0.05). This statistically significant difference confirms the efficacy of the HFT technique. The HFT applied in our research is effective for the correction of severe rigid nonidiopathic scoliosis, further correcting spinal deformity, which remarkably decreases large Cobb angles, greatly simplifies intraoperative operation difficulty, reduces surgical trauma, and reduces the overall risk of patients.

The complications related to HFT included pin loosening, superficial and deep pin tract infections, brain abscess, cerebral nerve damage, and brachial plexus injury. In our study, 1 patient suffered from superficial pin tract infections, and after debridement and anti-infection treatment, the patient recovered. One patient suffered from DVT and underwent inferior vena cava filter placement. Pin infection occurred in 1 patient and was controlled by debridement. Two patients developed a rigid knee/hip due to fear of moving the knee/hip, and the symptoms subsided after surgery. Traction with excessive weight, a long duration or a rapid increase in the load may lead to neurological complications. However, most of the neurologic injuries (92–100%) caused by preoperative traction were transient [19]. In our study, 1 patient had brachial plexus palsy, and 1 patient had femoral nerve palsy. Symptoms disappeared after removal of the increased weight. No patient developed neurological deficits when the surgical correction was finished. We believe that this is a benefit of preoperative traction, as the surgeons obtained an accurate assessment of the spinal cord status and then used this knowledge to soundly control cord tension. Meanwhile, it also explains why for extremely severe patients, we need preoperative traction instead of intraoperative traction alone. From the perspective of cord safety, we believe preoperative traction is far more relevant than intraoperative traction for this type of patient.

The limitations of this study were as follows: in this study, we focus on the improvement of coronal Cobb angle, in order to assess the contribution rate of strong HFT in the treatment of severe rigid non-idiopathic scoliosis. As for aspects including sagittal improvement and function recovery, further research is needed. Besides, the number of cases remains to be further accumulated.

## Conclusion

Strong halo-femoral traction plays a relatively significant role in the treatment of severe rigid nonidiopathic scoliosis while decreasing the risk of operation, and it is a safe and effective method for the treatment of severe rigid nonidiopathic scoliosis.

## Supplementary Information

**Additional file 1:** **Fig. S1.** Diagrammatic explanation to the terms used for the radiographic analysis.

## Data Availability

All data generated or analyzed during this study are included in this published article.

## Abbreviations

ACR: Absolute contribution rate
HFT: Halo-femoral traction
HGT: Halo-gravity traction
AP: Anteroposterior
MC: Major curve
RCR: Relative contribution rate
DVT: Deep vein thrombosis
