# Supplementary material for: Analysis of the corrective contribution of strong halo-femoral traction in the treatment of severe rigid nonidiopathic scoliosis
Source: J Orthop Surg Res. 2020 Nov 30;15:567. doi: 10.1186/s13018-020-02093-8 (PMC7706273; doi:10.1186/s13018-020-02093-8)
Supplement: Supplementary file 1 — Additional file 1: Fig. S1. Diagrammatic explanation to the terms used for the radiographic analysis. [file 13018_2020_2093_MOESM1_ESM.docx]

**Supplemental Figure**

**Supplemental Figure 1**


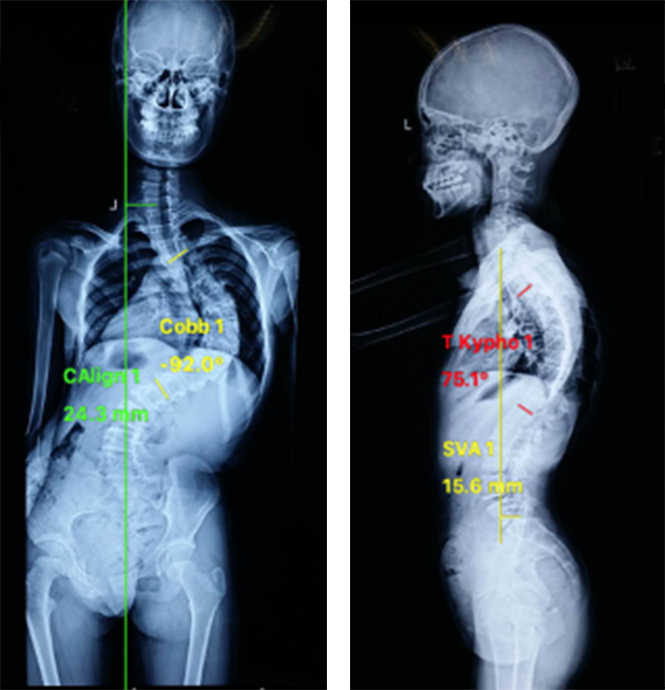


Supplemental Figure 1: Diagrammatic explanation to the terms used for the radiographic analysis.
